# A System of Surgery; Pathological, Diagnostic, Therapeutic, and Operative

**Published:** 1860-04

**Authors:** 


### EDITORIAL AND MISCELLANEOUS.

### Bibliographical.

*A System of Surgery; Pathological, Diagnostic, Therapeutic, and Operative.* By Samuel D. Gross, M. D., Professor of Surgery in the Jefferson Medical College, &c., &c.; illustrated with nine hundred and thirty-six engravings. 2 vol. 8 vo. Blanchard & Lea, Philadelphia, 1859.

This long looked for and much needed work has at last made its appearance in two portly volumes of near twelve hundred pages each. Every American heart should feel proud of this great production, as it will effectually silence our foreign detractors. We can now, with pride and pleasure, point them to this great work by the distinguished Professor of Surgery in the Jefferson Medical College—a work which will certainly favorably compare with the boasted productions of their most eminent Surgeons. From a careful examination of these volumes we feel that we have abundant cause to congratulate the profession upon the valuable addition, not only to the literature of Surgery, but their source of information upon this important branch of Medicine. The work is well adapted to a large class of the profession, and more particularly those who must necessarily limit themselves to a few books on surgery. The work embraces an extended field, there being no topic properly belonging to this branch of the medical science, that is not discussed. The following extract from the preface will give a better idea than we could possibly do, of the object and aim of the author in presenting this great work:

"The object of this work is to furnish a systematic and comprehensive treatise on the science and practice of surgery, considered in the broadest sense; one that shall serve the practitioner as a faithful and available guide in his daily routine of duty. It has been too much the custom of modern writers on this department of the healing art to omit certain topics altogether, and to speak of others at undue length, evidently assuming that their readers could readily supply the deficiencies from other sources, or that what has been thus slighted is of no particular practical value. My aim has been to embrace the whole domain of Surgery, and to allot to every subject its legitimate claim to notice in the great family of external diseases and accidents. How far this object has been accomplished, it is not for me to determine. It may safely be affirmed, however, that there is no topic, properly appertaining to surgery, that will not be found to be discussed, to a greater or less extent, in these volumes."

But notwithstanding we have said thus much (and we feel we have not said too much) in praise of this voluminous treatise, still we must be permitted to add, before proceeding further, that it is not as perfect as we would have it. Among other things, we feel that in several instances justice has not been done Surgeons of our own country. While some who have made valuable contributions to this department of medicine, are almost entirely ignored, others who merely reflect the brilliancy of their discoveries, are made prominent.—Why this is so, we will not pretend to say, but feel that it is much to be regretted, as this great work is now, and will be for years to come, the record or reference book to American Surgery. We do not, nor would we attribute anything improper to the distinguished author. That it is human to err, no one will deny.

The work is divided into two parts, one entitled *general* Surgery, and the other *special* Surgery, or diseases and injuries of particular organs, textures and regions. The first part occupies 676 pages of the first volume, and is devoted as above suggested, to the discussion of general principles. The remainder of the first volume and the whole of the second, making between seventeen and eighteen hundred pages, is devoted to *special* Surgery.

The discussion of inflammation and its results, occupies two hundred and fifty pages of the first part. We were pleased to see so large a space given to this important subject, as we entirely agree with the author as to the paramount importance of the great principles of Surgery. We are forced, too, to admit the humiliating fact that there is no subject so little understood by the general practitioner, as inflammation and its results. In introducing this important subject, the author makes the following truthful remarks :

"A thorough knowledge of inflammation is indispensable to every practitioner of Surgery. It should form the principal subject of his studies during his pupilage, and the main object of his professional contemplation in after life. When it is recollected that there is hardly any disease which comes within the province of this department of science, that does not originate in inflammation, or that is not more or less affected by it during its progress, the truth and force of these remarks will appear sufficiently obvious. The smallest pimple upon the nose is, in point of fact, as much an inflammation as an erysipelas that covers the face and head. An ulcer of one of the mucous follicles of the mouth, does not differ, in principle, from an ulcer of one of the glands of Peyer, which are the seat of so much disease and danger in typhoid fever. Many of the maladies, vaguely called nervous, are nothing but forms of inflammation, the nature and seat of which it is often difficult, and frequently impossible to determine. Their predominant symptoms are of a nervous character, and hence the diseases which they accompany, are usually considered as nervous, while in reality the reverse is too often the case.

All accidents, whatever may be their nature or degree, are necessarily followed, if the patient survive their immediate effects, by inflammation. The little wound made in venesection, the incision left in cupping, and the bite inflicted in leeching, would never heal without the aid of this process; the parts would remain open, and be the seat of incessant bleeding, or they would become festering and putrid sores. In a word, there would be no reparation after injuries of any kind, however simple, and operative surgery, instead of bearing healing on its wings, and being a blessing to our race, would be the merest cold-blooded butchery. Thus, it will be perceived that inflammation is capable of playing, as it were, a double game in the animal economy, being at one time a cause of death, and at another a source of life. It is for this reason that it is often designated by the terms healthy and unhealthy, according as the one or the other of these states predominates."

We next have a very interesting chapter on congenital malformations, in which the author arranges the various malformations under the following heads:

1st, from deficiency of parts ; 2ndly, redundancy of parts; 3rdly, displacement; 4thly, occlusion; 5thly, deviation of position; 6thly, adhesion of contiguous surfaces; 7thly, vascular tumours.

We have then in succession, a chapter upon the following subjects: Tumours, or morbid growths, including malignant disease, scrofula; general consideration and management of wounds; effect of injuries upon the nervous system; syphilis, and general diagnosis. Next comes the principles of operative surgery, with a chapter devoted to each of the following heads: minor surgery, principles of operative surgery, plastic surgery, subcutaneous surgery, amputations in general, and excision of the bones and joints. This division of the work concludes with a chapter on anesthetics or the means of averting pain.

We very much regret that our limited space will not permit us to give a more extended notice of this part of the work. We would be pleased to give the views of the author upon some of the points of doctrine and practice which are not considered as positively settled, but for the above reason must content ourself by saying that the subjects introduced are admirably arranged, and most thoroughly discussed.

With our limited space, we can do little more than merely mention the more prominent divisions of the seventeen hundred pages constituting the *second* part, or special Surgery. In the first chapter we have discussed various diseases and injuries of the skin and cellulo-adipose tissue, as elephantiasis, keloid, lepoid, authrax, burns, frost-bite, &c., &c.—The next three chapters are devoted to the diseases and injuries of muscles, tendons, lymphatic vessels, ganglions, and nerves. The succeeding two hundred pages are occupied in a discussion of the various injuries and diseases of the vasculor system. The author reviews, in a masterly manner, the various plans proposed for the cure of aneurism and his deductions are certainly most rational.

We are pleased to see fractures and dislocations occupy so prominent a place in this inestimable work. Near three hundred pages are devoted alone to these two injuries. In the general consideration of fractures, the author makes use of the following language: "If I were called upon to testify under oath, what branch of Surgery I regarded as most trying and difficult to practice successfully and creditably, I should unhesitatingly assert that it was that which relates to the present subject, (fracture) and I am quite sure that every enlightened practitioner would concur with me in the justice of this opinion." If this feeling was more general, we would not have to deplore that inefficiency in the treatment of this accident that all are forced to admit does at present exist in the profession.

The chapter, of an hundred or more pages, on the disease and injuries of the eye, is certainly one of the very best treatises for the student or general practitioner. He has divested the diseases of this important organ of their barbarous nomenclature, which we regard as the first step towards a more general understanding of this important class of affections.

In the discussion of the diseases and injuries of the female genital organs, we find vesico-vaginal fistula occupying a prominent place, but were pained to see that Dr. J. Marion Sims, to whom all honor is due for the present perfection in the treatment of this unpleasant accident, is placed entirely in the back-ground, and another made prominent, who, in my humble opinion, deserves no such position. It is true that Dr. Bozeman has suggested a new plan of fastening the sutures, as many others have done, but whether this should entitle him to all the honor of the present perfection in the treatment of this lesion, is not to be discussed. From a careful examination of the chapter upon this subject, no one would, for a moment, suppose that Dr. Sims had made the valuable contribution to this class of affections, that the distinguished author must himself admit, he has. We again repeat, that we were pained to see this injustice to one to whom the profession are under so many obligations for valuable contributions, and in a work, too, that we value so highly.

In bringing this hasty notice to a close, we feel we would be acting in bad faith did we not express our admiration of the style in which the work is published. The Publishers, Messrs. Blanchard & Lea, have certainly added new laurels to their well-earned reputation.

